# Biomarkers of endothelial glycocalyx dysfunction in pregnancy: a systematic review of clinical relevance and detection techniques

**DOI:** 10.1007/s00011-026-02208-7

**Published:** 2026-03-17

**Authors:** Federica Fogacci, Duha Yahya, Jeanine Roeters Van Lennep, Katariina Öörni, Valentina Di Micoli, Francesco De Seta, Cristina Scollo, Claudio Borghi, Arrigo Francesco Giuseppe Cicero

**Affiliations:** 1 Hypertension and Cardiovascular Risk Research Center, Medical and Surgical Sciences Department, Alma Mater Studiorum University of Bologna, 40130 Bologna, Italy; 2 Department of Medical Pharmacology, Medical Faculty, Ataturk University, 25240 Erzurum, Turkey; 3 International School of Medicine, Istanbul Medipol University, Istanbul, Turkey; 4 Department of Internal Medicine, Erasmus MC Cardiovascular Institute, University Medical Center Rotterdam, Rotterdam, The Netherlands; 5 Wihuri Research Institute, Helsinki, Finland; 6 Molecular and Integrative Biosciences Research Programme, Faculty of Biological and Environmental Sciences, University of Helsinki, Helsinki, Finland; 7 Department of Obstetrics and Gynecology, IRCCS San Raffaele Scientific Institute, University Vita and Salute, 20132 Milan, Italy; 8 Cardiovascular Medicine Unit, Heart, Chest and Vascular Department, IRCCS Azienda Ospedaliero-Universitaria di Bologna, 40138 Bologna, Italy

**Keywords:** Endothelial glycocalyx, Endothelial dysfunction, Pregnancy, Syndecan-1, Hyaluronic acid, Heparan sulfate, Gestational diabetes

## Abstract

**Objective and design:**

The endothelial glycocalyx (EG) is a key regulator of vascular homeostasis, acting as a dynamic barrier between the bloodstream and the endothelium. In pregnancy, structural and functional alterations of the EG have been increasingly implicated in the pathogenesis of endothelial dysfunction, particularly in preeclampsia and other vascular complications. This systematic review critically examines current evidence on circulating biomarkers of EG degradation and their clinical relevance in hypertensive and metabolic disorders of pregnancy.

**Methods:**

We explore the mechanistic role of the glycocalyx in maintaining vascular integrity, evaluate state-of-the-art detection methods—including sidestream dark field (SDF) imaging and biochemical assays—and summarize data on key circulating components such as syndecan-1, hyaluronic acid, heparan sulfate, and adhesion molecules.

**Results:**

Particular attention is given to distinguishing early- from late-onset preeclampsia and to other high-risk obstetric conditions, including gestational diabetes, fetal growth restriction, and infection-related complications. Despite heterogeneity across studies, most findings support a consistent association between EG disruption and adverse maternal–fetal outcomes.

**Conclusions:**

This review highlights the potential of glycocalyx-derived biomarkers and imaging tools as non-invasive indicators of microvascular injury. Their integration into existing surveillance models could enhance early risk stratification and open new avenues for targeted clinical interventions in cardio-obstetric care.

**Supplementary Information:**

The online version contains supplementary material available at 10.1007/s00011-026-02208-7.

## Introduction

Hypertensive disorders of pregnancy (HDP)—including gestational hypertension, preeclampsia, and chronic hypertension with superimposed preeclampsia—are among the leading causes of maternal and perinatal morbidity and mortality worldwide [1]. Preeclampsia, in particular, affects approximately 5–8% of pregnancies and is associated with serious complications such as preterm birth, intrauterine growth restriction, placental abruption, and multi-organ dysfunction [2]. Clinically, preeclampsia is typically classified according to gestational age at presentation as preterm (before 37 weeks of gestation), term (at or beyond 37 weeks), or postpartum (occurring from 48 h to 6 days after delivery) [3, 4]. Based on the timing of onset, preeclampsia is further categorized into early-onset (EOP), which occurs before 34 weeks of gestation, and late-onset (LOP), occurring at or after 34 weeks. It is now widely accepted that these two forms may differ in their underlying etiology and should be considered distinct phenotypes of the disease [5, 6].

Despite decades of research, the precise pathophysiological mechanisms underlying HDP remain incompletely understood. However, one consistently identified feature is widespread endothelial dysfunction, which is now recognized as a central component in the development and progression of these conditions [7].

Within this framework of vascular pathology, increasing attention has been directed toward the endothelial glycocalyx (EG)—a highly specialized, carbohydrate-rich layer that coats the luminal surface of endothelial cells [8]. This dynamic structure is composed of membrane-bound proteoglycans (e.g., syndecans, glypicans), glycosaminoglycans (e.g., heparan sulfate (HS), chondroitin sulfate, hyaluronic acid (HA)), glycoproteins, and various adsorbed plasma constituents [9]. Under physiological conditions, the EG serves as a mechanosensor and barrier, regulating vascular permeability, shear stress transduction, leukocyte adhesion, and coagulation [10–13]. It restricts the passage of macromolecules (> 70 kDa), cationic particles, and pathogenic agents such as bacteria and viruses, playing a crucial role in preserving endothelial integrity and systemic vascular homeostasis [14].

The glycocalyx is continuously remodeled in response to hemodynamic shear forces and enzymatic activity. It is sensitive to oxidative stress, inflammatory cytokines, ischemia–reperfusion injury, and hormonal fluctuations—all of which are prominent features of hypertensive pregnancy disorders. In preeclampsia, both structural degradation and functional impairment of the EG have been documented, particularly in early-onset cases [15]. This disruption contributes to increased vascular permeability, promotes the expression of adhesion molecules, facilitates leukocyte and platelet adhesion, and fosters a procoagulant and proinflammatory milieu [16]. Additionally, EG degradation reduces nitric oxide bioavailability, impairing vasodilation and exacerbating hypertension and end-organ damage [17]. Thus, glycocalyx injury may not simply be a consequence of endothelial dysfunction, but rather a potential upstream contributor to disease pathogenesis [18].

Technological advances have enabled the study of the EG in both experimental and clinical settings. Sidestream dark field (SDF) imaging provides a non-invasive, real-time method for evaluating microvascular EG thickness through quantification of the perfused boundary region (PBR) [19]. An increased PBR reflects glycocalyx thinning, which has been correlated with microvascular rarefaction and dysfunction in preeclampsia [20]. Biochemical techniques, including enzyme-linked immunosorbent assays (ELISA), high-performance liquid chromatography coupled with mass spectrometry (HPLC–MS/MS), and multiplex immunoassays, permit the quantification of EG-derived components in plasma or serum [21]. Among these, syndecan-1 (SDC1), HS, and HA have emerged as leading candidates for the detection of systemic glycocalyx shedding [22].

In non-obstetric settings, such as sepsis, trauma, and cardiovascular disease, elevated circulating levels of these markers have been associated with disease severity, adverse outcomes, and mortality [23–26]. Emerging evidence suggests a similar role in the context of pregnancy. Several studies have demonstrated increased concentrations of SDC1, HS, and HA in women with preeclampsia compared to normotensive controls, with higher levels observed in more severe or early-onset forms of the disease [22, 27]. Moreover, these markers have been associated with alterations in microcirculatory parameters and endothelial activation, as evidenced by elevated levels of adhesion molecules such as VCAM-1, ICAM-1, and E-selectin [28].

Despite these promising findings, the literature remains fragmented. There is considerable heterogeneity in study design, biomarker selection, analytical methodology, and clinical endpoints. Some investigations report strong associations between biomarker levels and disease severity, while others fail to find statistically significant differences [15, 29]. The clinical utility of these biomarkers in differentiating between early- and late-onset preeclampsia, predicting adverse outcomes, or guiding patient management also remains unclear. Furthermore, the dual maternal and placental origin of certain biomarkers, such as SDC1, complicates their interpretation and limits their specificity [30].

Given the complexity of EG biology and the multifactorial nature of HDP, a systematic synthesis of the available evidence is essential. In addition to clarifying the diagnostic and prognostic value of these biomarkers, such a review can identify methodological gaps, guide future research priorities, and explore the potential of EG markers as targets for therapeutic intervention. Indeed, if validated, these biomarkers could facilitate early detection of endothelial injury, enabling risk stratification, closer surveillance, and timely clinical interventions. They may also inform the development of novel therapeutic strategies aimed at preserving or restoring glycocalyx integrity—such as antioxidants, statins, or synthetic glycocalyx mimetics [31].

To our knowledge, no comprehensive systematic review has specifically addressed the use of circulating laboratory biomarkers of EG degradation in pregnant women with hypertensive disorders. The objective of this review, therefore, is to identify, evaluate, and synthesize existing clinical studies investigating such biomarkers in the context of HDP. We aim to assess the range of markers studied, their association with disease subtypes and severity, and their potential diagnostic and prognostic utility. In doing so, we hope to lay the groundwork for future biomarker-driven approaches in maternal–fetal medicine and to enhance our understanding of vascular pathophysiology in hypertensive pregnancy disorders.

## Methods

### Study design and registration

This systematic review was conducted in accordance with the Preferred Reporting Items for Systematic Reviews and Meta-Analyses (PRISMA) 2020 guidelines [32] and was prospectively registered in the PROSPERO database (CRD42023114227).

### Literature search strategy

A comprehensive literature search was performed using PubMed and Scopus, covering studies published from database inception through May 2025. The search strategy included combinations of controlled vocabulary and free-text terms related to the EG and pregnancy-specific conditions, such as *“endothelial glycocalyx” AND (pregnancy OR preeclampsia OR gestational diabetes OR fetal growth restriction)*. No restrictions were placed on publication language, study design, or geographic origin in order to maximize sensitivity and inclusiveness. The full, database-specific search strategies are detailed in Supplementary File S1. Reference lists of included articles and relevant reviews were also screened to identify additional eligible studies not captured in the initial search.

### Eligibility criteria

Studies were eligible for inclusion if they met the following criteria: they involved pregnant women as the study population and included a direct or indirect assessment of EG integrity. Eligible assessment methods comprised non-invasive imaging techniques (such as PBR measurements obtained through SDF imaging), biochemical analysis of glycocalyx components (including SDC1, HA, and HS), or ultrastructural evaluation via electron microscopy.

Studies were excluded if they were non-original research, including narrative or systematic reviews, editorials, conference abstracts, or case reports. Additional exclusion criteria included animal studies and any study that did not report pregnancy-specific outcomes related to EG structure or function.

### Study selection

The selection process was conducted in two stages. First, the titles and abstracts of all retrieved records were independently screened by two reviewers to assess potential eligibility. Full-text articles were subsequently obtained for studies deemed potentially relevant. Any discrepancies or disagreements between reviewers regarding inclusion were resolved through discussion and consensus; if necessary, a third reviewer was consulted to achieve resolution.

### Data extraction

Two reviewers independently extracted data using a standardized sheet. Extracted variables included study design, population characteristics, biomarker types, analytical methods, and key outcomes. Discrepancies were resolved through discussion.

### Risk of bias assessment

The methodological quality of included studies was assessed using the Newcastle–Ottawa Scale (NOS), which is specifically designed for evaluating the risk of bias in cohort and case–control studies [33]. Each study was independently evaluated by two reviewers based on the NOS criteria, which consider selection of study groups, comparability, and outcome or exposure assessment. Disagreements in scoring were resolved through consensus, and when necessary, adjudicated by a third reviewer.

### Data synthesis

Due to substantial heterogeneity in biomarker types, analytical methods, timing of assessment, and clinical contexts among the included studies, a meta-analysis was deemed inappropriate. Instead, a descriptive narrative synthesis was performed. Extracted data were systematically categorized by obstetric complication—including preeclampsia, gestational diabetes mellitus, fetal growth restriction, and infection-related conditions. Quantitative findings, such as PBR values and circulating biomarker concentrations, were summarized using descriptive statistics, including means with standard deviations and medians with interquartile ranges. Where applicable, data were presented in tabular form to facilitate direct comparison across studies.

## Study selection and characteristics

The initial database search yielded 52 records. After removal of duplicates and screening of titles and abstracts, a total of 16 clinical studies met the inclusion criteria and were included in this review. A PRISMA flow diagram detailing the selection process is provided in Supplementary File S2.

All included studies were original clinical investigations involving pregnant women, with either direct or indirect assessment of EG structure or function. The total sample size across studies was 1,784 participants, with individual study populations ranging from 27 to 188 subjects. Most studies employed a case–control or cross-sectional design, while a smaller subset incorporated prospective elements.

The majority of studies focused on preeclampsia, either comparing early- and late-onset phenotypes or assessing biomarker levels in relation to disease severity. Several studies also evaluated EG biomarkers in other obstetric conditions, including gestational diabetes mellitus (GDM), fetal growth restriction (FGR), superimposed preeclampsia in women with chronic kidney disease (CKD), and infection-related complications such as acute pyelonephritis.

A range of biomarkers associated with glycocalyx integrity were investigated, with SDC1, HA, and heparan sulfate proteoglycans (HSPG) being the most frequently reported. Additional analytes included vascular adhesion molecules (e.g., VCAM-1, ICAM-1, selectins), glycosaminoglycans (e.g., dermatan sulfate, keratan sulfate), and fibronectin. Biomarkers were primarily measured using enzyme-linked immunosorbent assay (ELISA), although some studies employed more advanced techniques such as HPLC–MS/MS or Luminex multiplex platforms.

Only a limited number of studies utilized non-invasive imaging to assess glycocalyx morphology in vivo, most notably sidestream dark field (SDF) imaging to measure the perfused boundary region (PBR) as a surrogate of glycocalyx thickness. One study also incorporated electron microscopy to directly evaluate glycocalyx ultrastructure in omental tissue samples.

Overall, the included studies displayed substantial heterogeneity in terms of clinical populations, biomarker panels, timing of sample collection, and definitions of disease subtypes. Despite this variability, most studies reported a trend toward increased glycocalyx degradation or biomarker shedding in association with hypertensive or inflammatory obstetric complications. A detailed overview of the study populations, biomarkers assessed, analytical methods, and principal findings is provided in Table 1.

**Table 1:** Summary of clinical studies investigating biomarkers of endothelial glycocalyx dysfunction in pregnancy

References	Country	Study Population	Sample Size	Biomarkers Assessed	Analytical Techniques	Key Findings
Austgulen et al. [34]	Norway	PE, normotensive pregnant women, non-pregnant women	80 (25 PE, 40 NP, 15 NPW)	E-selectin, ICAM-1, VCAM-1	ELISA	Adhesion molecules increased in PE; no difference between normotensive pregnant and non-pregnant women
Bramham et al. [35]	UK	Pregnant women with chronic hypertension (with/without superimposed PE), controls	Cohort study: 180 (90 CH, 90 NP); Nested Case–control study: 93 (17 CH + PE, 76 CH)	SDC1	ELISA	SDC1 levels at 26–27 + 6 weeks were lower in those who developed superimposed PE
Dogan et al. [36]	Turkey	Early-onset PE, late-onset PE, normotensive controls	160 (43 EOP, 37 LOP, 80 NP)	Fibronectin, VCAM-1	ELISA	Fibronectin and sVCAM-1 levels were elevated in early-onset PE vs. controls
Hassani Lahsinoui et al. [27]	Netherlands	PE (± HELLP), NP	125 (65 PE, 60 NP)	DS, HS, KS, SDC1	ELISA; HPLC–MS/MS	DS increased and KS decreased in PE; no difference in SDC1 or HS
Immonen et al. [37]	USA	Acute pyelonephritis, normotensive pregnancy, non-pregnant women	188 (33 APN, 130 NP, 25 NPW)	SDC1	ELISA	SDC1 levels increased with gestational age; highest in pyelonephritis with bacteremia
Juusela et al. [30]	USA	SGA vs. AGA pregnancies	180 (50 SGA, 130 AGA)	SDC1	ELISA	SDC1 levels were lower in SGA pregnancies with abnormal Doppler; potential marker of placental dysfunction
Kim et al. [38]	Korea	Normal pregnancy, mild PE, severe PE	178 (63 NP, 33 mild PE, 82 severe PE)	E-selectin, ICAM-1, VCAM-1	ELISA	All biomarkers elevated in PE; VCAM-1 levels distinguished severity
Kornacki et al. [29]	Poland	Early-onset PE, late-onset PE, normotensive controls	60 (20 per group)	HA, VCAM-1	ELISA	HA and sVCAM-1 elevated in both PE subtypes vs. controls; no difference between EOP and LOP
Kornacki et al. [39]	Poland	Early-onset PE, late-onset PE, normotensive controls	60 (20 per group)	HA, SDC1	ELISA	HA elevated in PE vs. controls; SDC1 levels lower in PE vs. controls; no difference between PE subtypes
Long et al. [40]	New Zealand	Pregnant women at 20 weeks (who developed GDM vs. controls)	40 (20 GDM, 20 NP)	CS, HA, HS, SDC1	ELISA	No significant predictive value of any biomarker for GDM onset
Mugerli et al. [41]	Slovenia	Severe PE, term NP, non-pregnant women	27 (9 per group)	EG (structural)	Transmission EM	EG thickness significantly reduced in PE compared to both control groups
Rios et al. [42]	Brazil	Severe PE vs. NP	120 (60 per group)	ICAM-1, VCAM-1	ELISA	VCAM-1 elevated in PE; no difference between early- and late-onset PE
Watanabe et al. [43]	Japan	Uncomplicated pregnancies, PE, gestational hypertension	81 (22 UP, 36 PE, 23 GH)	HA, SDC1	ELISA	HA levels were highest in PE, followed by GH and controls; SDC1 was lower in both PE and GH vs. controls
Weissgerber et al. [15]	USA	Normotensive pregnancies, early-onset PE, late-onset PE, GDM	73 (14 EOP, 29 LOP, 21 GDM, 9 NP)	HA, HSPG, PBR, SDC1	SDF imaging; ELISA	PBR, HA, and HSPG elevated in early-onset PE; SDC1 correlated with gestational age
Wiles et al. [44]	UK	CKD with or without superimposed PE, PE, NP	120 (15 CKD with superimposed PE, 45 CKD, 18 PE, 20 NP, 22 NPW)	E-selectin, HA, P-selectin, VCAM-1	ELISA; Luminex	HA and VCAM-1 significantly higher in CKD with superimposed PE
Ziganshina et al. [45]	Russia	Early-onset PE, late-onset PE, NP	60 (20 EOP, 20 LOP, 20 NP)	HA, SDC1	ELISA	HA elevated in PE vs. controls; SDC1 lower in PE vs. controls; no difference between PE subtypes

AGA: Appropriate for Gestational Age; APN: Acute Pyelonephritis; CH: Chronic Hypertension; CS: Chondroitin Sulfate; CKD: Chronic Kidney Disease; DS: Dermatan Sulfate; EG: Endothelial Glycocalyx; ELISA: Enzyme-Linked Immunosorbent Assay; EOP: Early-Onset Preeclampsia; EM: Electron Microscopy; FGR: Fetal Growth Restriction; GDM: Gestational Diabetes Mellitus; GH: Gestational Hypertension; HA: Hyaluronic Acid; HS: Heparan Sulfate; HSPG: Heparan Sulfate Proteoglycans; ICAM-1: Intercellular Adhesion Molecule 1; KS: Keratan Sulfate; LOP: Late-Onset Preeclampsia; NP: Normotensive Pregnant Women; NPW Non-Pregnant Women; PE: Preeclampsia; PBR: Perfused Boundary Region; SDC1: Syndecan-1; SGA: Small for Gestational Age; SDF: Sidestream Dark Field; VCAM-1: Vascular Cell Adhesion Molecule 1

## Risk of bias assessment

The methodological quality of the included studies was assessed using the NOS, as detailed in the Methods section. Overall, the studies demonstrated moderate to high quality, with NOS scores ranging from 5 to 9 out of a possible 9. Most investigations scored well in the selection and outcome/exposure domains, particularly in clearly defining the study population and employing validated biomarker measurement techniques. However, greater variability emerged in the comparability domain, reflecting inconsistent adjustment for confounding variables such as gestational age, comorbidities, or mode of delivery. While several studies implemented multivariate analyses or group-matching strategies, others failed to report whether key confounders were controlled for, thereby reducing internal validity. Additionally, a small subset of studies lacked explicit descriptions of blinding, either in outcome assessment or biomarker analysis, which may have introduced detection bias. This limitation was particularly noted in observational cohorts involving heterogeneous pregnancy complications (e.g., pyelonephritis, CKD, SGA) where potential clinical overlap could influence interpretation. Despite these limitations, the overall methodological rigor was acceptable for synthesis, and no study was excluded solely on the basis of high risk of bias. Full details of individual study scores, along with domain-specific evaluations, are provided in Supplementary File S3.

## Structure and biochemical composition of the endothelial glycocalyx

**Fig. 1:**
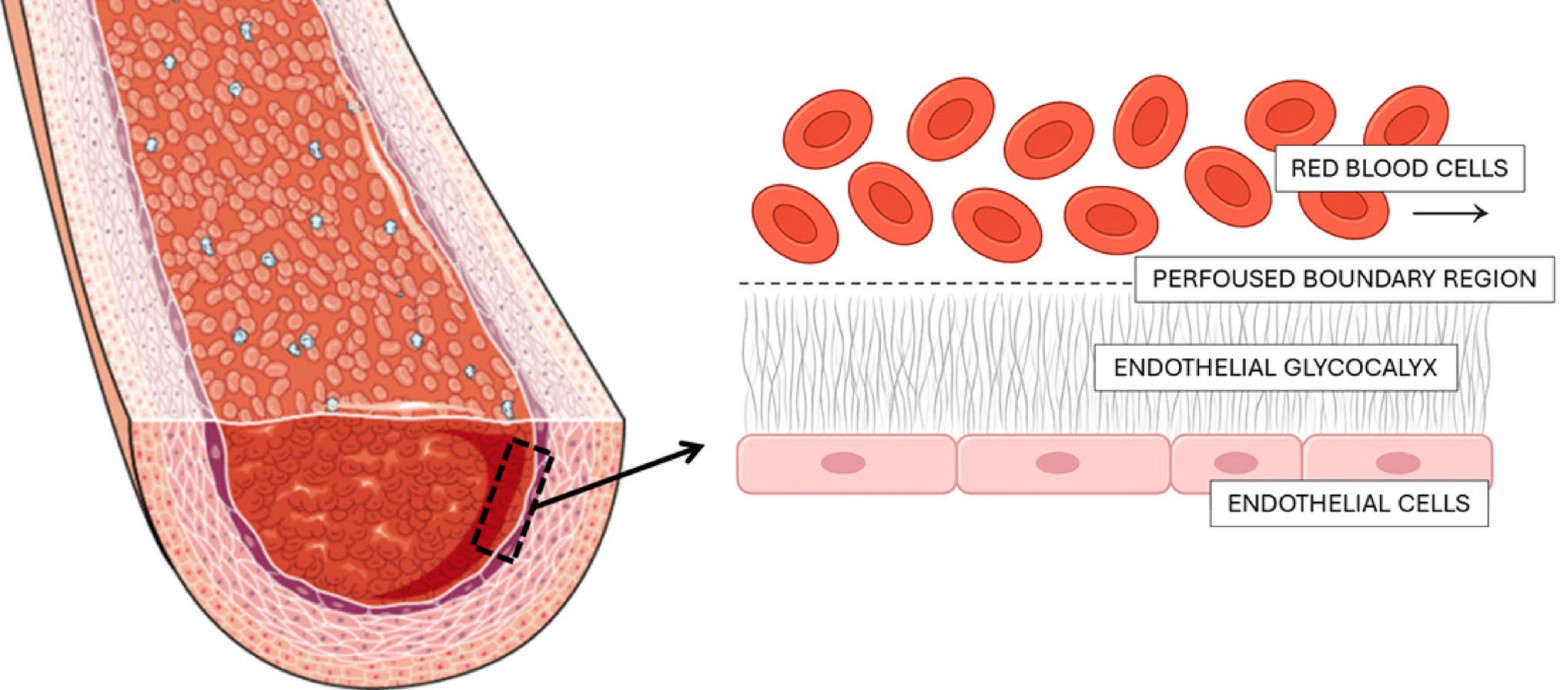
Microvascular structure and measurement regions. Schematic cross-section of a capillary showing red blood cells within the lumen. The endothelial glycocalyx overlays the endothelium (pink cells), and the dashed line indicates the perfused boundary region

The EG is a gel-like, carbohydrate-rich matrix that coats the luminal surface of endothelial cells and is integral to the maintenance of vascular homeostasis [46]. Its architecture comprises a dense network of membrane-bound proteoglycans and glycoproteins interwoven with soluble plasma- and endothelium-derived components [47]. This structural complexity, as schematically represented in Fig. 1, highlights the position of the EG between the bloodstream and the endothelium, where it acts simultaneously as a molecular sieve and a mechanotransducer [48].

### Structural organization

The glycocalyx is anchored to the endothelial cell membrane via core proteins such as syndecans and glypicans, which form the backbone for glycosaminoglycan (GAG) side chains [49]. These GAGs—HS, chondroitin sulfate, dermatan sulfate, keratan sulfate, and HA—determine the glycocalyx’s electrostatic properties and binding affinities [50]. The highly sulfated nature of many GAGs imparts a strong negative charge, which not only restricts the penetration of circulating macromolecules and contributes to selective vascular permeability [51], but also promotes interactions with positively charged molecules.

The glycocalyx is not a static barrier but a dynamic structure in a constant state of synthesis and degradation [52]. Its composition and thickness fluctuate in response to enzymatic activity (e.g., heparanase, hyaluronidase, matrix metalloproteinases) and hemodynamic forces, such as shear stress. This turnover complicates its geometric definition, as visualized in vivo or ex vivo [53].

Electron microscopy and intravital imaging studies have revealed that the glycocalyx forms a dense, three-dimensional meshwork that extends into the vascular lumen [54, 55]. The outermost extent of red blood cell (RBC) penetration into this structure defines the perfused boundary region (PBR), a measurable parameter inversely proportional to glycocalyx thickness [56]. A higher PBR reflects a compromised glycocalyx and has been associated with early atherosclerosis, albuminuria, and HDP [57, 58].

### Proteoglycans

Proteoglycans are the primary structural components of the glycocalyx, consisting of a core protein covalently linked to one or more GAG chains [59]. Key proteoglycans in the endothelium include syndecans (particularly syndecan-1), glypicans, perlecan, and decorin. These proteins are heterogeneous in size and GAG content, which can vary according to cellular context and environmental stimuli [46].

HS is the most abundant GAG in the EG and plays a pivotal role in ligand binding, signal transduction, and vascular permeability. Enzymatic modifications of saccharide units within GAG chains, particularly sulfation patterns, fine-tune these interactions and modulate the functional properties of the glycocalyx [60].

### Glycoproteins

Glycoproteins serve to anchor the glycocalyx to the endothelial cell surface and include numerous adhesion molecules with well-characterized roles in immune cell recruitment and vascular inflammation [61]. Among these, E-selectin and P-selectin mediate initial leukocyte tethering, while integrins (e.g., αVβ3, α2β1) and immunoglobulin superfamily members such as ICAM-1, VCAM-1, and PECAM-1 promote firm adhesion and transmigration [60].

Other glycoproteins embedded within the glycocalyx contribute to hemostasis and thromboregulation, such as the GPIb-IX-V complex, which plays a role in platelet adhesion. These proteins, while structurally distinct from proteoglycans, are critical for mediating the glycocalyx’s barrier and signaling functions [60].

### Soluble components

Interspersed within the proteoglycan-glycoprotein scaffold are soluble plasma proteins such as albumin and orosomucoid, which enhance the charge selectivity and structural integrity of the glycocalyx. These components can modulate local viscosity, buffer ionic gradients, and contribute to colloid osmotic pressure at the endothelial surface [60].

While some of these molecules are passively incorporated, others bind specifically to GAG motifs and participate in physiological processes such as inflammation, coagulation, and vascular permeability. Their displacement or loss into the circulation is a hallmark of glycocalyx shedding and vascular dysfunction [62].

## Assessment of glycocalyx integrity

A range of complementary methodologies has been developed to investigate EG status in vivo [63], spanning imaging-based, biochemical, and ultrastructural approaches.

### Sidestream dark field (SDF) imaging

SDF imaging enables non-invasive, real-time evaluation of sublingual microcirculation [64, 65]. Through quantification of the PBR, this method indirectly reflects EG thickness. Under normal conditions, a thick glycocalyx impedes RBC penetration, resulting in a lower PBR. In contrast, a degraded glycocalyx permits deeper RBC ingress, reflected by a higher PBR value.

Commercial systems, such as the GlycoCheck software coupled with the CapiScope Handheld Video Capillaroscopy System, provide semi-automated analysis [56], and have been validated in both physiological and pathological contexts, including sepsis, diabetes, and preeclampsia [15, 66, 67].

### Biochemical detection in blood samples

Quantitative assessment of glycocalyx degradation products in blood samples complements imaging data by revealing the systemic biochemical footprint of endothelial injury [68]. Circulating levels of syndecan-1, HS proteoglycans (HSPG), and HA can be measured using ELISAs. These markers reflect both maternal and, in some cases, placental endothelial integrity [69]. It should be noted that most commercially available HSPG ELISA kits are designed to detect perlecan (HSPG2), which is only one member of the HSPG family. Some studies, however, do not specify which kit was used, creating uncertainty as to whether the reported values represent perlecan specifically or total HSPGs.

Advanced analytical techniques such as high-performance liquid chromatography coupled with tandem mass spectrometry (HPLC–MS/MS) and Luminex-based multiplex assays allow simultaneous quantification of multiple biomarkers with high specificity and sensitivity [70]. These include soluble adhesion molecules (VCAM-1, ICAM-1, E-selectin, P-selectin), GAGs (dermatan sulfate, keratan sulfate), and inflammatory mediators (Table 2).

**Table 2:** Laboratory biomarkers of endothelial glycocalyx dysfunction in pregnancy and associated detection techniques. Detection methods include ELISA, HPLC–MS/MS, and Luminex FlexMap3D platforms

Biomarker	Detection Techniques	Clinical Associations	Strength of Association
Chondroitin Sulfate (CS) [40]	ELISA	Not associated with the subsequent development of GDM	Weak / Inconsistent
Dermatan Sulfate (DS) [27]	HPLC–MS/MS	Significantly elevated in women with preeclampsia compared to normotensive pregnant controls	Moderate
E-Selectin [38]	ELISA, Luminex	Increased in both mild and severe preeclampsia vs. controls; no difference between mild and severe PE	Moderate
Heparan Sulfate [27, 40]	HPLC–MS/MS (Waters Quattro Premier XE)	Heparan sulfate levels did not differ significantly between preeclamptic and normotensive women before delivery; not associated with GDM	Weak / Inconsistent
Heparan Sulfate Proteoglycans (HSPG) [15]	ELISA	Significantly increased in early-onset preeclampsia vs. both normotensive pregnant women	Moderate
Hyaluronic Acid (HA) [29, 40, 44]	ELISA	Elevated in both early- and late-onset preeclampsia compared to controls, not significantly different between PE subtypes. Also elevated in CKD with superimposed PE. Not predictive of GDM	Strong
ICAM-1 [34, 37, 38, 42]	ELISA	Elevated in preeclampsia, especially in severe forms. Also increased in acute pyelonephritis	Moderate
Keratan Sulfate (KS) [27]	HPLC–MS/MS	Significantly decreased in preeclamptic women compared with normotensive pregnant controls	Moderate
L-Selectin [37]	ELISA	Reduced levels observed in preeclamptic pregnancies compared to normotensive controls	Weak / Inconsistent
PECAM-1 [37]	ELISA	No significant differences reported between preeclamptic and normotensive pregnant groups	Weak / Inconsistent
P-Selectin [44]	ELISA, Luminex	Median levels higher in preeclampsia; no diagnostic value for superimposed PE	Weak / Inconsistent
Soluble VCAM-1 (sVCAM-1) [29, 42]	ELISA	Elevated in severe PE compared to normotensive pregnancies; no significant difference between early- and late-onset PE	Moderate
Syndecan-1 (SDC1) [35, 37, 40]	ELISA	Positively correlated with gestational age. Levels elevated during pregnancy and further increased in acute pyelonephritis, particularly with bacteremia. No association with GDM. Lower levels may reflect placental dysfunction in growth-restricted or hypertensive pregnancies	Weak / Inconsistent
VCAM-1 [37, 38]	ELISA, Luminex	Elevated in both mild and severe preeclampsia; higher levels in severe forms. Also increased in acute pyelonephritis	Moderate

AGA: Appropriate for Gestational Age; CKD: Chronic Kidney Disease; CS: Chondroitin Sulfate; DS: Dermatan Sulfate; ELISA: Enzyme-Linked Immunosorbent Assay; GAGs: Glycosaminoglycans; GDM: Gestational Diabetes Mellitus; HA: Hyaluronic Acid; HPLC–MS: High-Performance Liquid Chromatography-Mass Spectrometry; HSPG: Heparan Sulfate Proteoglycans; ICAM-1: Intercellular Adhesion Molecule 1; KS: Keratan Sulfate; MS: Mass Spectrometry; PE: Preeclampsia; SDC1: Syndecan-1; VCAM-1: Vascular Cell Adhesion Molecule 1

### Histological and ultrastructural analysis

Transmission electron microscopy (TEM), though invasive and restricted to biopsy specimens, remains the gold standard for direct visualization of the glycocalyx ultrastructure [71]. Perfusion fixation with agents such as ruthenium red enables the detailed morphometric assessment of glycocalyx area, density, and continuity [72]. This method has revealed marked reductions in glycocalyx coverage in omental biopsies from patients with severe preeclampsia, offering anatomical confirmation of systemic endothelial injury [41].

## Diagnostic implications

### Clinical relevance of glycocalyx biomarkers in preeclampsia

Preeclampsia is a leading complication of pregnancy, affecting up to 8% of gestations worldwide [73]. It is characterized by new-onset hypertension accompanied by signs of maternal organ dysfunction, frequently coexisting with fetal growth restriction [74]. Increasing evidence points to degradation of the EG as a key pathological feature of both early- and late-onset forms of the disease [45].

Non-invasive assessments using SDF imaging have consistently demonstrated elevated PBR values in women with preeclampsia, reflecting glycocalyx thinning and impaired microvascular function [15]. In early-onset cases, these structural alterations are accompanied by reductions in capillary perfusion and elevated circulating levels of glycocalyx-associated components such as syndecan-1, hyaluronic acid, and HSPG —indicating systemic endothelial injury [15].

Long-term vascular sequelae have also been observed [75]. Elevated PBR measurements have been reported even one year postpartum in women with a history of preeclampsia, suggesting persistent microvascular dysfunction beyond pregnancy [76].

Circulating adhesion molecules—including VCAM-1, ICAM-1, and E-selectin—are consistently found at higher concentrations in preeclamptic pregnancies, with levels correlating with disease severity [38]. These markers show a stepwise increase from mild to severe clinical phenotypes and may reflect the extent of endothelial activation and inflammatory recruitment [38]. By contrast, data on SDC1 are less consistent. Some studies report elevated levels in preeclampsia, while others find no significant difference compared to normotensive controls at term [77, 78]. Such discrepancies may stem from variations in sampling timing, disease stage, or the dual maternal and placental origin of the biomarker.

### Comparison between early- and late-onset preeclampsia

Efforts to differentiate EOP from LOP based on the extent of EG injury have yielded mixed results. In some studies, serum levels of HA and VCAM-1 were similarly elevated in both EOP and LOP groups when compared to normotensive pregnancies, suggesting a common underlying mechanism of endothelial dysfunction regardless of gestational age at onset. Other investigations, however, have indicated a more severe vascular insult in early-onset disease [79]. In particular, significantly higher concentrations of HA and HSPG were observed in women with EOP, pointing to more extensive glycocalyx disruption in the earlier manifestation of the disorder [45].

### Glycocalyx biomarkers in other pregnancy-associated disorders

Beyond preeclampsia, EG disruption has been explored in a range of other obstetric conditions, particularly in GDM, SGA, and autoimmune disorders. In these contexts, EG impairment appears to be primarily mediated by endothelial stress, inflammation, and oxidative damage. Nevertheless, direct evidence remains limited, with few well-designed clinical studies establishing clear associations between EG biomarkers and these complications. Further research into EG dynamics may offer valuable insights and open new avenues for diagnostic or therapeutic interventions.

In pregnancies complicated by SGA fetuses—particularly those with abnormal umbilical and uterine Doppler velocimetry—lower circulating levels of SDC1 have been observed [30]. This reduction may reflect compromised placental vascular integrity and an association with fetal growth restriction.

Conversely, elevated SDC1 levels have been detected in cases of acute pyelonephritis, especially when accompanied by bacteremia, indicating that glycocalyx shedding may also serve as a marker of systemic endothelial activation in infection-related pregnancy complications [37].

The potential of glycocalyx-derived biomarkers to predict GDM has also been investigated [40]. However, measurements of SDC1, HA, HS, and chondroitin sulfate at mid-gestation did not demonstrate predictive value for the later development of GDM, suggesting limited utility of these markers in this context [40].

### Histological evidence of glycocalyx loss

Ultrastructural evidence of glycocalyx degradation has been confirmed through TEM of omental biopsies [42]. In patients with severe preeclampsia, a marked reduction in glycocalyx area was observed compared to both normotensive pregnant and non-pregnant controls [15]. These findings provide direct histopathological support for the biochemical and microvascular alterations associated with the disease, reinforcing the concept of systemic endothelial injury as a central feature of its pathophysiology.

## Discussion

This systematic review highlights the pivotal role of EG degradation in the pathophysiology of preeclampsia and other pregnancy-related disorders. Both structural alterations, detectable through imaging modalities such as SDF videomicroscopy, and biochemical evidence of glycocalyx shedding, reflected by elevated circulating biomarkers, converge to support the hypothesis that EG disruption is not merely a consequence, but may contribute to—and amplify— a driving force in the development of endothelial dysfunction during pregnancy. These approaches offer distinct yet complementary diagnostic perspectives.

SDF imaging provides a real-time, non-invasive assessment of EG thickness [80]. An elevated PBR reflects the penetration of red blood cells into the normally cell-free glycocalyx zone and has been consistently associated with pathological states such as preeclampsia, both in its early- and late-onset forms [15]. The method has shown high reproducibility across multiple studies and holds promise as a bedside-compatible tool for microvascular assessment in obstetric patients. Its ability to detect subclinical endothelial damage may be particularly valuable in the early identification of women at risk, enabling preemptive monitoring and intervention [81].

In parallel, circulating biomarkers of glycocalyx degradation—particularly SDC1, HA, and HS—offer complementary insights. These molecules can enter the circulation following enzymatic cleavage and/or shear stress–induced shedding of the EG and serve as biochemical fingerprints of systemic endothelial injury [82]. Nevertheless, circulating glycocalyx-related biomarkers may primarily reflect the degree of endothelial activation/injury—potentially as a secondary manifestation of preeclampsia—rather than providing direct evidence of a primary causal mechanism. Among them, SDC1 has been the most extensively studied, although its interpretative value remains challenged by variability across studies [83]. Factors such as gestational age at sampling, assay methodology, and the combined maternal and placental contribution of SDC1 contribute to heterogeneity in the reported findings. Conversely, HA and HS have demonstrated more consistent elevations in preeclamptic pregnancies, particularly in early-onset cases, and may be less influenced by gestational age and placental contribution, although their tissue sources and specificity for EG injury remain important considerations [84].

Biomarkers of endothelial activation, such as VCAM-1, ICAM-1, and E-selectin, are also frequently elevated in preeclampsia and further support the inflammatory and pro-adhesive phenotype characteristic of glycocalyx disruption [85]. While these molecules do not derive directly from the EG, their presence in circulation reflects downstream consequences of glycocalyx loss, such as increased leukocyte adhesion and endothelial permeability [86].

A critical consideration is the comparative utility of imaging-based versus biochemical approaches. PBR measurement provides a direct, functional indicator of EG integrity in the microcirculation, offering spatial resolution and the ability to detect subtle physiological changes that precede overt endothelial injury. It is particularly well suited for real-time monitoring, longitudinal assessment, and studies seeking to correlate microvascular parameters with clinical trajectories.

In contrast, circulating biomarkers are more accessible in standard clinical workflows and offer systemic information, potentially capturing widespread endothelial stress beyond a localized vascular bed. They are especially useful when imaging is not feasible—such as in resource-limited settings, in late gestation when patient cooperation may be limited, or in retrospective studies based on stored biological samples.

Taken together, these tools are complementary rather than redundant. PBR may be more appropriate for early detection, risk stratification, and physiological monitoring, while plasma biomarkers may aid in diagnostic confirmation, severity grading, and prognostication. The integration of both approaches may enhance diagnostic precision and open avenues for multimodal risk modeling in maternal–fetal medicine.

Despite the growing body of evidence, several challenges still hinder the immediate clinical application of these biomarkers. There remains a pressing need to standardize sampling protocols, assay platforms, and establish gestational age-specific reference ranges. Furthermore, distinguishing between maternal and placental sources of glycocalyx components—especially SDC1—is crucial to improve both the specificity and interpretability of these markers. This is particularly relevant for SDC1, whose circulating levels may reflect a combination of maternal endothelial shedding and placental release [22]. Accordingly, until studies are specifically designed to disentangle these sources, the mechanistic interpretation of circulating glycocalyx markers as primary drivers of disease will remain inherently limited. Moreover, most studies to date are observational and cross-sectional, which further limits our ability to draw conclusions about temporal dynamics or causality.

While SDF microscopy provides valuable morphological insights into the microvasculature, it offers limited information on the functional status of the glycocalyx. The technique is also time-intensive and relatively expensive, which may restrict its widespread clinical adoption. These limitations highlight the urgent need for methodological improvements and greater automation to enhance both feasibility and reproducibility.

Importantly, glycocalyx injury appears to play a role beyond preeclampsia. Associations have been observed with conditions such as fetal growth restriction, gestational diabetes, and infection-related pregnancy complications, suggesting that glycocalyx degradation may be a common vascular denominator across multiple obstetric syndromes. This opens the door to the possibility of incorporating endothelial glycocalyx assessment into broader prenatal monitoring, especially for high-risk pregnancies.

Moving forward, research should focus on validating the diagnostic and prognostic value of glycocalyx biomarkers in large, multicenter cohorts, as well as exploring their integration with established clinical tools like angiogenic profiling and Doppler velocimetry. Additionally, therapeutic approaches aimed at protecting or restoring glycocalyx integrity—such as antioxidant supplementation, statin therapy, or synthetic glycocalyx mimetics—deserve thorough investigation in the context of pregnancy.

### Translational perspectives and future directions

Emerging insights into the role of endothelial glycocalyx injury in hypertensive and metabolic complications of pregnancy offer a promising basis for translational innovation. Moving beyond association studies, future research must prioritize the clinical implementation of glycocalyx assessment through standardization of sampling protocols, development of gestational age-specific reference ranges, and validation in large, diverse cohorts. Additionally, integrating glycocalyx-derived biomarkers with existing maternal–fetal risk stratification tools—such as angiogenic profiles or uterine artery Doppler indices—may enhance predictive modeling and facilitate personalized antenatal care pathways.

Another critical frontier lies in exploring therapeutic modulation of the glycocalyx. Targeted interventions aimed at stabilizing or restoring glycocalyx integrity, including non-anticoagulant heparinoids, statins, or synthetic glycocalyx mimetics, warrant evaluation in pregnancy-specific settings. Such strategies may offer vascular protection without compromising placental perfusion or fetal development.

Moreover, although our review focuses primarily on the diagnostic and pathophysiological role of biomarkers in preeclampsia, their potential relevance for predicting long-term cardiovascular outcomes should also be considered. Women with a history of preeclampsia face a two- to four-fold increased risk of chronic hypertension, metabolic syndrome, and atherosclerotic cardiovascular disease later in life [87, 88]. Biomarkers of endothelial dysfunction, angiogenic imbalance (e.g., sFlt-1, PlGF), inflammation, and glycocalyx degradation may thus provide early signals not only of pregnancy-related vascular complications but also of persistent vascular vulnerability after delivery. If validated in longitudinal cohorts, such profiles could refine cardiovascular risk stratification in this high-risk population and enable timely preventive interventions.

Ultimately, the transition from biomarker discovery to clinical application will require interdisciplinary collaboration across obstetrics, vascular biology, and translational medicine. This approach aligns with the 2025 ESC Guidelines, which recommend the establishment of Pregnancy Heart Teams to provide coordinated care for women at elevated cardiovascular risk throughout pre-conception, pregnancy, and postpartum periods [89]. By anchoring glycocalyx research within a clinically actionable framework, there is significant potential to transform vascular assessment in pregnancy from a retrospective diagnosis to a proactive, preventive paradigm.

**Table 3:** Hypothetical clinical algorithm integrating endothelial glycocalyx assessment for risk stratification in pregnancy

Step	Clinical Action	Tools Involved	Interpretation / Thresholds	Suggested Next Steps
1	Identify at-risk pregnancies	Maternal history, Doppler ultrasound	Risk factors: chronic hypertension, prior PE, abnormal uterine flow	Proceed to biomarker testing
2	First-tier glycocalyx biomarker screening	Serum HA, HSPG (± sFlt-1/PlGF)	Elevated levels vs. gestational age-matched norms	Consider EG imaging if positive
3	Functional glycocalyx imaging	SDF imaging → PBR measurement	PBR > gestational norm (e.g., > 2.5 μm) suggests glycocalyx thinning	Stratify as “vascular high risk”
4	Integrative risk assessment	Combine biomarkers + imaging + angiogenic/Doppler profiles	Concordant abnormalities across ≥ 2 modalities	Intensify monitoring, consider early intervention
5	Clinical application	Tailored surveillance	Based on individualized vascular profile	Timing of delivery, hospitalization

This model is conceptual and intended to highlight the translational potential of glycocalyx assessment. Thresholds should be validated in prospective studies before clinical implementation

BP: blood pressure; CKD: chronic kidney disease; Doppler: Doppler velocimetry; EG: endothelial glycocalyx; HA: hyaluronic acid; HSPG: heparan sulfate proteoglycans; PBR: perfused boundary region; PE: preeclampsia; PlGF: placental growth factor; SDF: sidestream dark field; sFlt-1: soluble fms-like tyrosine kinase-1

### Toward a multimodal clinical algorithm

Translating glycocalyx research into actionable clinical pathways remains a key challenge in cardio-obstetric medicine. Given the multifactorial nature of endothelial dysfunction in pregnancy, no single marker is likely to suffice for early risk identification. We propose a tiered, multimodal approach in which biochemical and imaging-based assessments of the endothelial glycocalyx are integrated into existing surveillance strategies for high-risk pregnancies.

The first step would involve targeted biomarker screening—for instance, measurement of circulating HA or HSPG in the second trimester—applied to women with established risk factors such as chronic hypertension, abnormal uterine Doppler flow, or prior early-onset preeclampsia. In cases where biomarkers exceed gestational reference ranges, non-invasive microvascular imaging (e.g., sidestream dark field videomicroscopy with perfused boundary region quantification) could serve as a second-tier diagnostic tool to functionally assess glycocalyx integrity.

Patients exhibiting abnormalities across both dimensions—biochemical and functional—may be stratified as "vascular high-risk" and considered for intensified surveillance protocols, including more frequent blood pressure monitoring, fetal growth assessments, or early initiation of preventive strategies. This concept is consistent with the 2025 ESC Guidelines, which recommend that women classified as WHO 2.0 class II–III or above receive collaborative management by a Pregnancy Heart Team, ensuring comprehensive risk assessment, individualized care planning, and continuous monitoring throughout pregnancy and postpartum [89]. In the long term, this model could facilitate individualized management algorithms, inform trial eligibility for vascular-protective therapies, and potentially optimize the timing of delivery in borderline cases.

**Fig. 2:**
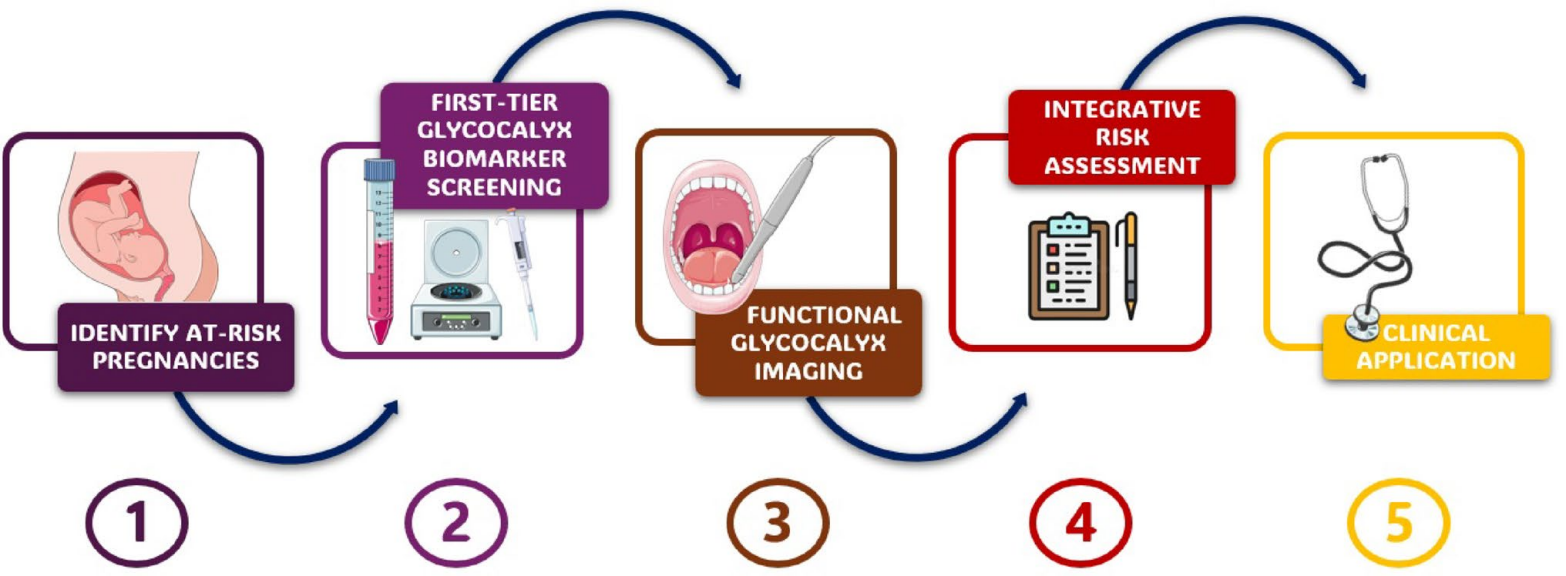
Multimodal algorithm for glycocalyx-based risk stratification. Conceptual flowchart illustrating a tiered approach integrating biomarker screening and microvascular imaging to identify high-risk pregnancies and guide individualized surveillance in cardio-obstetric care

Integration of glycocalyx-derived biomarkers into clinical pathways should be embedded within the framework of the Pregnancy Heart Team to ensure coordinated risk assessment, surveillance, and individualized interventions. The conceptual structure of the proposed algorithm is represented in the flowchart in Fig. 2, whereas an analytical description of its steps is reported in Table 3.

While investigational, such an approach illustrates the potential of endothelial glycocalyx profiling to bridge the current gap between mechanistic insight and clinical utility in maternal–fetal care.

## Conclusions

In conclusion, the EG represents a promising target for advancing our understanding, diagnosis, and management of hypertensive and vascular disorders in pregnancy. The integration of functional imaging techniques with circulating biomarkers offers a powerful platform for translational research, with the potential to shift vascular assessment in pregnancy from a reactive to a proactive approach.

## Supplementary Information

Below is the link to the electronic supplementary material.

Supplementary Material 1

Supplementary Material 2

Supplementary Material 3

## Data Availability

All data generated or analysed during this study are included in this published article and its supplementary information files.
