# Supplementary material for: Biomarkers of endothelial glycocalyx dysfunction in pregnancy: a systematic review of clinical relevance and detection techniques
Source: Inflamm Res. 2026 Mar 17;75(1):47. doi: 10.1007/s00011-026-02208-7 (PMC12995992; doi:10.1007/s00011-026-02208-7)
Supplement: Supplementary file 1 — Supplementary Material 1 [file 11_2026_2208_MOESM1_ESM.docx]

**Supplementary File S2.** PRISMA 2020 Flow diagram of study selection.

**Identification of studies via databases and registers**

Records removed *before screening*:

Duplicate records removed (n = 16)

Records identified from Databases (n = 52)

**Identification**

Records screened

(n = 36)

Records excluded

(n = 20)

Reports sought for retrieval

(n = 16)

**Screening**

Reports assessed for eligibility

(n = 16)

Studies included in review

(n = 16)

Reports of included studies

(n = 16)

**Included**
