# Supplementary material for: Biomarkers of endothelial glycocalyx dysfunction in pregnancy: a systematic review of clinical relevance and detection techniques
Source: Inflamm Res. 2026 Mar 17;75(1):47. doi: 10.1007/s00011-026-02208-7 (PMC12995992; doi:10.1007/s00011-026-02208-7)
Supplement: Supplementary file 2 — Supplementary Material 2 [file 11_2026_2208_MOESM2_ESM.docx]

**Supplementary File S1.** Full Search Strategies by Database.

**1. PubMed (via MEDLINE)**

("endothelial glycocalyx"[MeSH Terms] OR "endothelial glycocalyx"[All Fields] OR "vascular glycocalyx"[All Fields]) AND ("pregnancy"[MeSH Terms] OR "pregnancy"[All Fields] OR "preeclampsia"[MeSH Terms] OR "preeclampsia"[All Fields] OR "gestational diabetes"[MeSH Terms] OR "gestational diabetes"[All Fields] OR "fetal growth restriction"[All Fields] OR "small for gestational age"[All Fields] OR "HELLP syndrome"[All Fields] OR "pyelonephritis"[All Fields]) AND ("biomarkers"[MeSH Terms] OR "biomarkers"[All Fields] OR "syndecan-1"[All Fields] OR "hyaluronic acid"[All Fields] OR "heparan sulfate"[All Fields] OR "adhesion molecules"[All Fields] OR "VCAM-1"[All Fields] OR "ICAM-1"[All Fields])

Filters applied: Humans
Language: None
Document type: All (excluded reviews during screening phase)

**2. Scopus**

TITLE-ABS-KEY("endothelial glycocalyx" OR "vascular glycocalyx") AND TITLE-ABS-KEY("pregnancy" OR "preeclampsia" OR "gestational diabetes" OR "fetal growth restriction" OR "SGA" OR "HELLP syndrome" OR "pyelonephritis") AND TITLE-ABS-KEY("biomarker" OR "syndecan-1" OR "hyaluronic acid" OR "heparan sulfate" OR "VCAM-1" OR "ICAM-1" OR "adhesion molecule")

Filters: Article type = Research articles
Language = English
Subject area = Medicine
