# Supplementary material for: Biomarkers of endothelial glycocalyx dysfunction in pregnancy: a systematic review of clinical relevance and detection techniques
Source: Inflamm Res. 2026 Mar 17;75(1):47. doi: 10.1007/s00011-026-02208-7 (PMC12995992; doi:10.1007/s00011-026-02208-7)

**Supplementary File S2.** Risk of bias assessment based on the Newcastle–Ottawa Scale. Most studies scored between 7 and 9, indicating overall moderate-to-high methodological quality.

| **First Author (Year)** | **Selection (0–4)** | **Comparability (0–2)** | **Outcome/Exposure (0–3)** | **Total Score (0–9)** |
| --- | --- | --- | --- | --- |
| Austgulen (1997) | 4 | 1 | 2 | 7 |
| Bramham (2020) | 4 | 2 | 2 | 8 |
| Dogan (2014) | 4 | 1 | 3 | 8 |
| Hassani Lahsinoui (2021) | 4 | 1 | 2 | 7 |
| Immonen (2023) | 4 | 1 | 3 | 8 |
| Juusela (2023) | 3 | 1 | 2 | 6 |
| Kim (2004) | 4 | 2 | 3 | 9 |
| Kornacki (2020) | 4 | 1 | 3 | 8 |
| Kornacki (2021) | 4 | 1 | 2 | 7 |
| Long (2016) | 3 | 1 | 2 | 6 |
| Mugerli (2022) | 4 | 1 | 3 | 8 |
| Rios (2015) | 4 | 1 | 3 | 8 |
| Watanabe (2023) | 4 | 2 | 2 | 8 |
| Weissgerber (2019) | 4 | 2 | 3 | 9 |
| Wiles (2019) | 4 | 2 | 3 | 9 |
| Ziganshina (2022) | 4 | 1 | 2 | 7 |


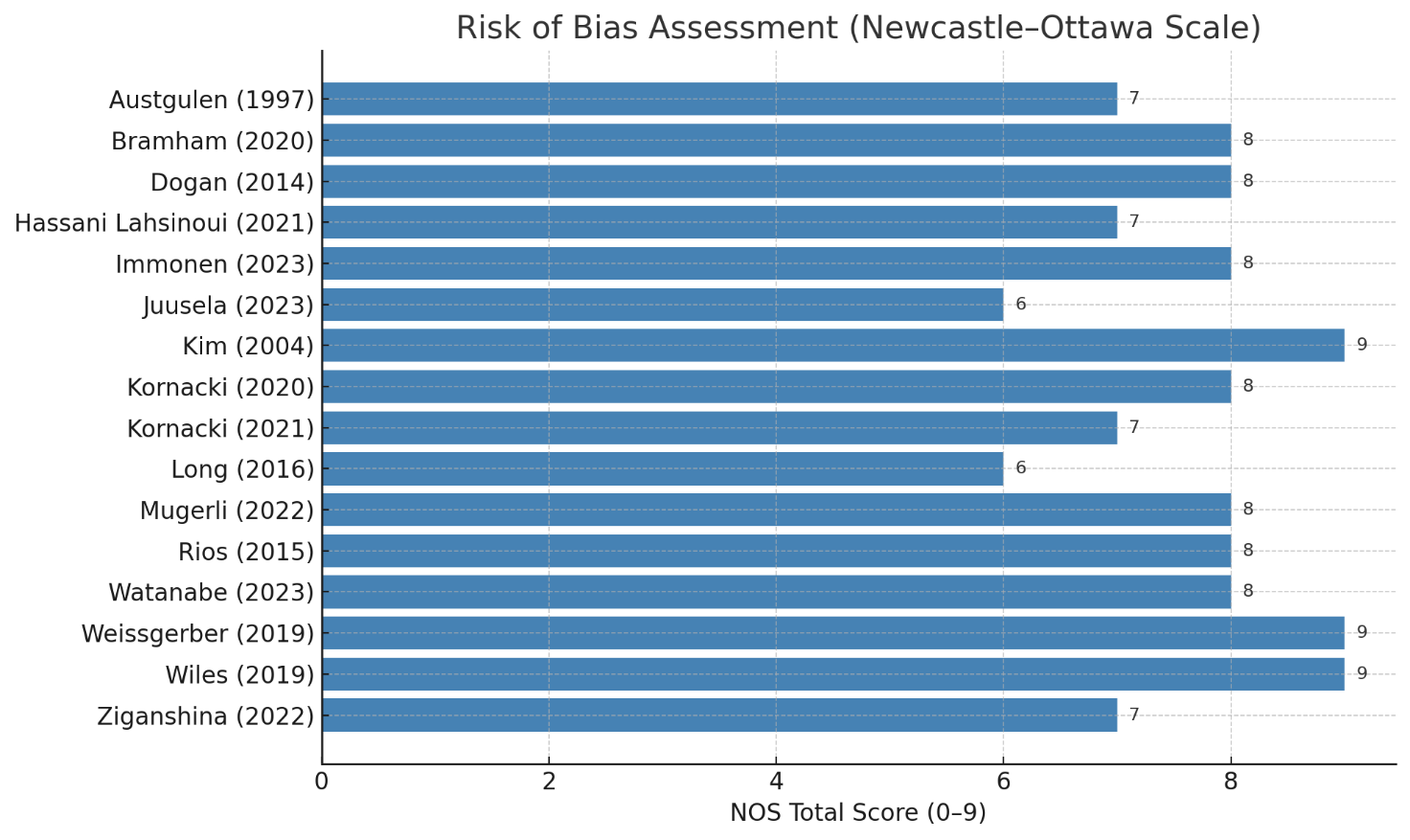

Supplement: Supplementary file 3 — Supplementary Material 3 [file 11_2026_2208_MOESM3_ESM.docx]
